# Surgical paradox: physiological activation and improved mood state during surgical work in pediatric surgeons

**DOI:** 10.1093/joccuh/uiag036

**Published:** 2026-07-04

**Authors:** Hisae Iida, Kazuto Suda, Ayaka Adachi, Michiaki Ikegami, Hiroko Watayo, Kentaro Fujiwara, Takamasa Suzuki, Kumpei Abe, Ailing Hu, Hiroyuki Kobayashi, Atsuyuki Yamataka, Go Miyano

**Affiliations:** Department of Pediatric General and Urogenital Surgery, Juntendo University School of Medicine, 2-1-1 Hongo, Bunkyo-ku, Tokyo 113-8421, Japan; Department of Pediatric General and Urogenital Surgery, Juntendo University School of Medicine, 2-1-1 Hongo, Bunkyo-ku, Tokyo 113-8421, Japan; Department of Pediatric General and Urogenital Surgery, Juntendo University School of Medicine, 2-1-1 Hongo, Bunkyo-ku, Tokyo 113-8421, Japan; Department of Pediatric General and Urogenital Surgery, Juntendo University School of Medicine, 2-1-1 Hongo, Bunkyo-ku, Tokyo 113-8421, Japan; Department of Pediatric General and Urogenital Surgery, Juntendo University School of Medicine, 2-1-1 Hongo, Bunkyo-ku, Tokyo 113-8421, Japan; Department of Pediatric General and Urogenital Surgery, Juntendo University School of Medicine, 2-1-1 Hongo, Bunkyo-ku, Tokyo 113-8421, Japan; Department of Pediatric General and Urogenital Surgery, Juntendo University School of Medicine, 2-1-1 Hongo, Bunkyo-ku, Tokyo 113-8421, Japan; Department of Pediatric General and Urogenital Surgery, Juntendo University School of Medicine, 2-1-1 Hongo, Bunkyo-ku, Tokyo 113-8421, Japan; Department of Personalized Kampo Medicine, Juntendo University Graduate School of Medicine, Tokyo, Japan; Department of Personalized Kampo Medicine, Juntendo University Graduate School of Medicine, Tokyo, Japan; Department of Pediatric General and Urogenital Surgery, Juntendo University School of Medicine, 2-1-1 Hongo, Bunkyo-ku, Tokyo 113-8421, Japan; Department of Pediatric General and Urogenital Surgery, Juntendo University School of Medicine, 2-1-1 Hongo, Bunkyo-ku, Tokyo 113-8421, Japan

**Keywords:** pediatric surgery, surgical stress, cortisol, autonomic nervous balance, POMS2, mood disturbance

## Abstract

**Objectives:**

Pediatric surgeons often experience physical and psychological strain. However, surgery may provide motivation and fulfillment. To explore this dual nature of surgical work, we examined physiological and psychological stress responses of pediatric surgeons on surgical working days (SWs) and nonsurgical working days (NSWs).

**Methods:**

Nineteen pediatric surgeons participated in this cross-sectional study. Salivary cortisol, autonomic nervous activity using a wearable device (low/high heart rate ratio [LHR]), and total mood disturbance (TMD) score, calculated from subscales of the Profile of Mood States 2nd Edition (POMS2) as an index of mood state, were assessed at 2 time points: before and after surgery by the operator on SWs (mean preoperative time: 11:16 am; mean postoperative time: 2:18 pm) and at 8:00 am and 5:00 pm on NSWs. Data were analyzed using mixed linear models and univariate tests to evaluate the influence of sex, post-call status, experience, and surgical site.

**Results:**

On both SWs and NSWs (54 and 44 evaluation sessions from 19 pediatric surgeons, respectively), the post-work decrease in cortisol levels was smaller on SWs than on NSWs (*P* < .05). LHR increased significantly after surgery (SWs) but not on NSWs (*P* < .05), indicating a sympathetically activated state, particularly remarkable in post-call status compared with non-post-call status (*P* < .05). TMD scores were lower on SWs than on NSWs (*P* < .05), reflecting improved mood during surgical activity. More experienced surgeons (>5 years) had lower TMD scores for both SWs and NSWs (*P* < .05).

**Conclusions:**

Despite physical and psychological demands, performing surgery was linked to reduced mood disturbance among pediatric surgeons.

## Teaser

**Key points**
 

- **What is already known on this topic:** Surgeons are exposed to substantial occupational stress, which has been associated with fatigue, reduced concentration, and potential risks to patient safety. Physiological indicators such as heart rate variability and salivary cortisol, along with psychological tools including POMS2, have been used to assess work-related stress. However, most studies have emphasized psychological distress and workload, with limited focus on positive aspects of surgeons’ psychological responses during surgical work.
- **What this study adds:** This study demonstrates that surgical working days are associated with sustained physiological activation, as indicated by a smaller decline in cortisol levels and sustained sympathetic dominance, while simultaneously being linked to improved psychological states reflected by lower total mood disturbance scores. Notably, younger surgeons showed greater mood improvement on surgical days.
- **How this study might affect research, practice, or policy:** These findings highlight the importance of considering both physiological strain and mood-state changes in occupational health research on surgeons. In practice, combined assessment of autonomic activity, cortisol, and mood may support early identification of work-related health risks. From a policy perspective, optimizing work organization to balance physiological burden with task-related activation may contribute to sustainable surgical practice.

## 1. Introduction

In health care professionals, excessive stress can lead to accumulated fatigue and decreased concentration, which may compromise the safety and quality of medical care.^1^^,^^2^ Therefore, appropriately assessing stress and translating such findings into workplace improvements are expected to contribute to safer health care delivery.^3^ Establishing reliable methods for stress assessment is crucial for surgeons, who inevitably experience chronic fatigue, and several studies have addressed its importance and methodology. For example, Weenk et al.^4^ monitored autonomic nervous balance intraoperatively using a wearable device and reported that fellows and residents exhibited greater sympathetic predominance than did consultants. Furthermore, Caitlin et al.^5^ demonstrated a sustained increase in surgeons’ heart rate over 2 days after being on-call, indicating chronic stress. In modern health care settings, effectively managing stress is a major challenge as it can influence not only the well-being of medical professionals but also the outcomes of the patients they treat. However, physiological activation during surgery may not always reflect detrimental stress alone. Interestingly, another study found that surgeons with higher sympathetic activation at the beginning of surgery had fewer postoperative complications in their patients,^6^ suggesting that physiological activation during surgery may also include adaptive or performance-related components. These findings indicate that surgical work may involve both physiological strain and adaptive task-related activation.

Recently, our group evaluated perioperative stress in pediatric patients using noninvasive measurements of salivary cortisol and autonomic balance monitored by a wearable device. We found that younger age, longer operation time, and undergoing abdominal surgery rather than surface or thoracic surgery were associated with higher stress levels.^7^ Moreover, postoperative cortisol levels correlated with the degree of pain, suggesting that these biomarkers can noninvasively reflect the perioperative status of pediatric patients. Most stress measurement studies in the medical field suggest their role for evaluating workload, workplace environment patrols, and patient stress monitoring. However, we also considered that surgeons, despite engaging in diverse tasks, may experience positive psychological responses while performing surgery and contributing to patient care. Therefore, we hypothesized that, beyond physical and psychological strain, surgical work may be associated with favorable mood-state changes.

In recent years, the Profile of Mood States 2nd Edition (POMS2) has been widely used as a self-administered questionnaire to evaluate transient mood states. It has been applied to quantify mood disturbances among caregivers and nurses working in intensive care units, as well as to assess the effects of interventions aimed at improving mental health.^8^^,^^9^ Although various studies have examined occupational stress among health care workers, few have specifically focused on surgeons during operative procedures, and few studies have evaluated mood-state changes during surgical work. Therefore, in the present study, we assessed the physical and psychological stress and mood fluctuations among pediatric surgeons during surgical work using salivary cortisol measurements, autonomic nervous balance monitoring via a wearable device, and POMS2-based mood assessments.

## 2. Material and methods

### 2.1 Study participants and design

**Figure 1:**
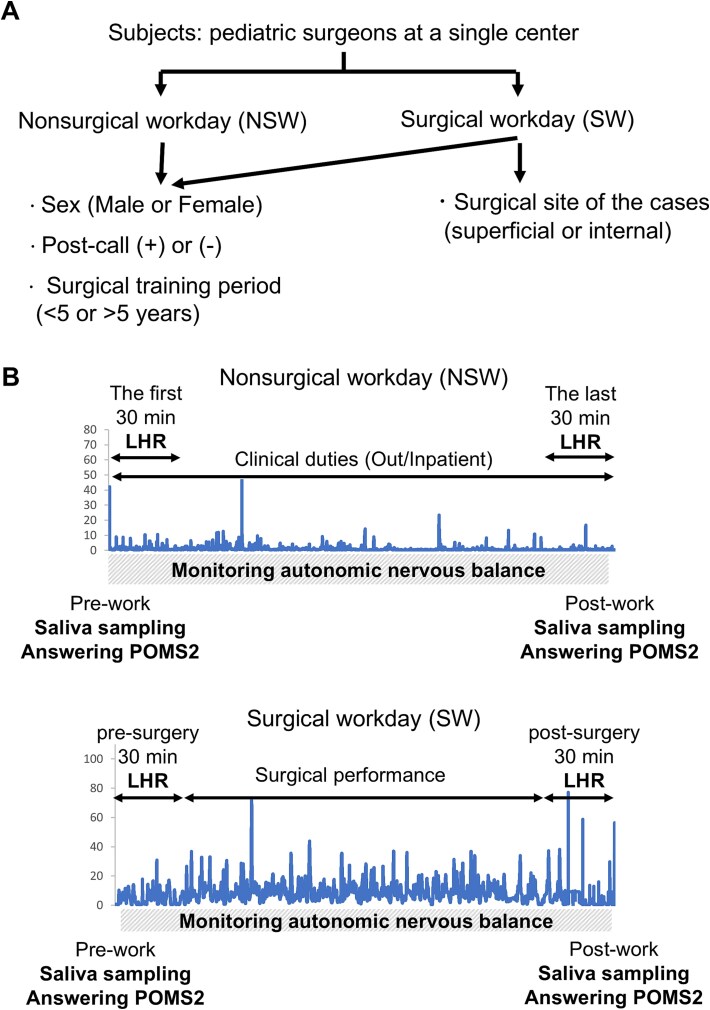
Study protocol and data collection. (A) Overview of the study schedule. Participants were categorized into the surgical working days (SW) and nonsurgical working days (NSW) groups. In addition, sex, on-call status on the previous day, years of surgical experience, and type of surgical case were evaluated as stratification factors. (B) Timeline of wearable device use, saliva sampling, and POMS2 questionnaire administration for SW and NSW groups. On NSWs, salivary sampling and POMS2 were performed before and after work, and autonomic nervous activity was monitored during working hours. On SWs, salivary sampling and POMS2 were performed 30 minutes pre-surgery and 30 minutes post-surgery, and autonomic nervous activity was monitored from 30 minutes pre-incision to 30 minutes post-closure. Mean LHR values were extracted for the first and last 30 minutes of the working hours on NSWs, and for the period from 30 minutes before incision to 30 minutes after closure on SWs. In addition, all continuously monitored LHR values obtained on each day in the NSW and SW groups were plotted and analyzed. LHR, low-/high-frequency heart rate ratio; POMS2, Profile of Mood States 2nd Edition.

This cross-sectional study was conducted among pediatric surgeons at different training levels, including fellows, board-certified surgeons, and supervising surgeons at a single center to examine their physical and psychological stress and mood state (disturbance) (Figure 1). Only participants whose data were collected on at least 2 occasions were included in the analysis. In this study, each evaluation session consisted of salivary cortisol sampling, autonomic nervous activity monitoring, and POMS2 assessment. Based on the surgeons’ work, the evaluation periods were categorized into 2 types: surgical workdays (SWs) and nonsurgical workdays (NSWs). In the SW group, saliva samples for cortisol measurement and responses to the POMS2 questionnaire for mood state assessment were collected 30 minutes before and after surgery. In the SW group, the mean (±SD) saliva sampling and POMS2 responses times were 11:16 am ±130 minutes preoperatively and 2:18 pm ± 168 minutes postoperatively. Autonomic nervous balance was monitored using a wearable device from 30 minutes pre-incision to 30 minutes post-closure for each surgical case. In the NSW group, saliva samples and POMS2 responses were obtained at 8:00 am and 5:00 pm, and autonomic nervous balance monitoring was conducted between 8:00 am and 5:00 pm.

Subgroup analyses according to sex (male or female), post-call status (post-call [+] or post-call [−]), and surgical training period (<5 years or *>*5 years) were conducted as exploratory analyses. Only for the SW group were the surgical cases categorized by the surgical site (superficial or internal procedure).

### 2.2 Saliva collection, sample processing, and cortisol measurement

Saliva was collected using commercially available saliva assay kits (Salimetrics LLC, USA) and age-appropriate techniques according to the manufacturer’s instructions. If samples could not be collected correctly for any reason, the subjects were excluded from this study. Saliva samples were processed as follows: at least 200 μL of saliva was extracted and frozen at −80°C following centrifugation at 1500 *g* for 15 minutes. In each sample, salivary cortisol was determined using a Salivary Cortisol Enzyme-Linked Immunosorbent Assay (ELISA) kit (Salimetrics LLC, USA) according to the manufacturer’s instructions.

### 2.3 Assessing autonomic reactivity

**Figure 2:**
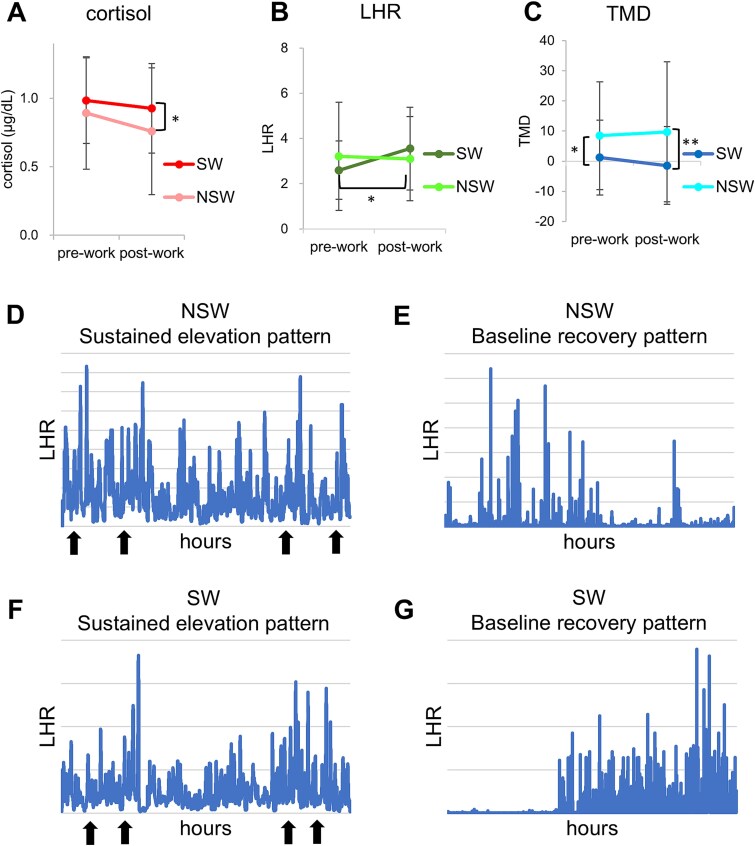
Comparison of salivary cortisol, LHR, and TMD between SW and NSW groups. (A) Pre- and post-work concentration of salivary cortisol. ^*^P < .05. (B) Pre- and post-work autonomic balance assessed by low-/high-frequency heart rate ratio (LHR). ^*^P < .05. (C) Pre- and post-work TMD scores obtained by POMS2. ^*^P < .05 and ^**^P < .01. (D, E) Examples of the sustained elevation pattern and the baseline recovery pattern of LHR in the NSW group. The arrows indicate representative periods in which LHR remained elevated without apparent recovery toward baseline. (F, G) Examples of the sustained elevation pattern and the baseline recovery pattern of LHR in the SW group. The arrows indicate the points where the LHR remains elevated for a period without returning to the baseline. LHR, low-/high-frequency heart rate ratio; NSW, nonsurgical working days; POMS2, Profile of Mood States 2nd Edition; SW, surgical working days; TMD, total mood disturbance.

**Figure 3:**
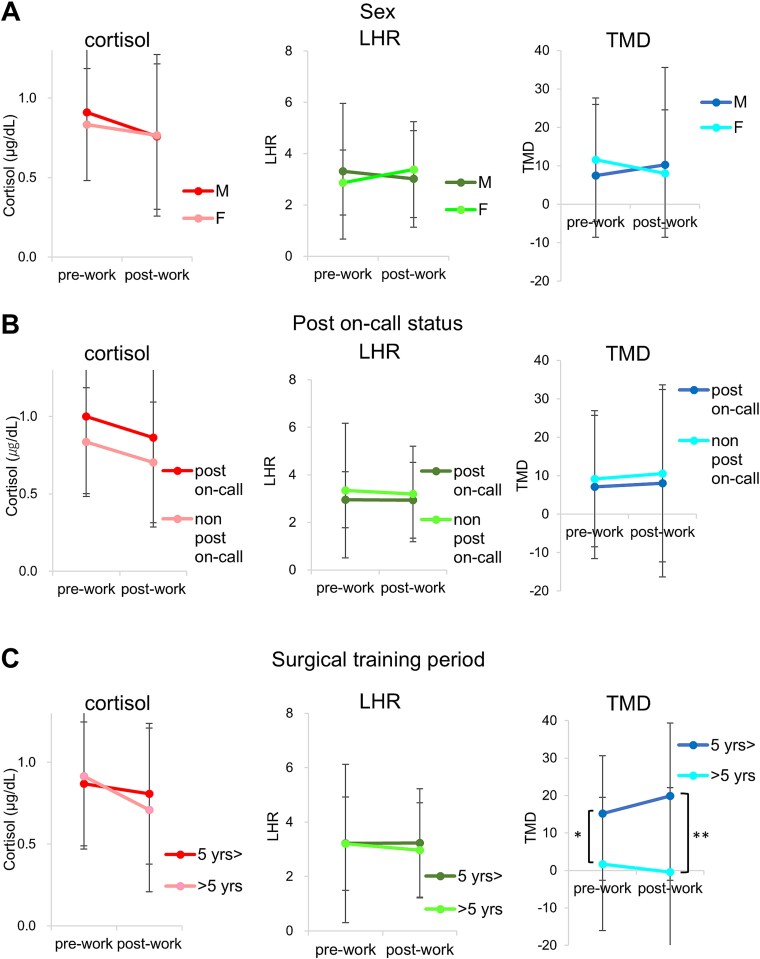
Changes in salivary cortisol, LHR, and TMD in the NSW group based on differences by sex, on-call status, and years of experience. (A) Comparison by sex. (B) Comparison by post-on-call status. (C) Comparison by surgical training period. ^*^P < .05, ^**^P < .01. LHR, low-/high-frequency heart rate ratio; NSW, nonsurgical working days; TMD, total mood disturbance.

**Figure 4:**
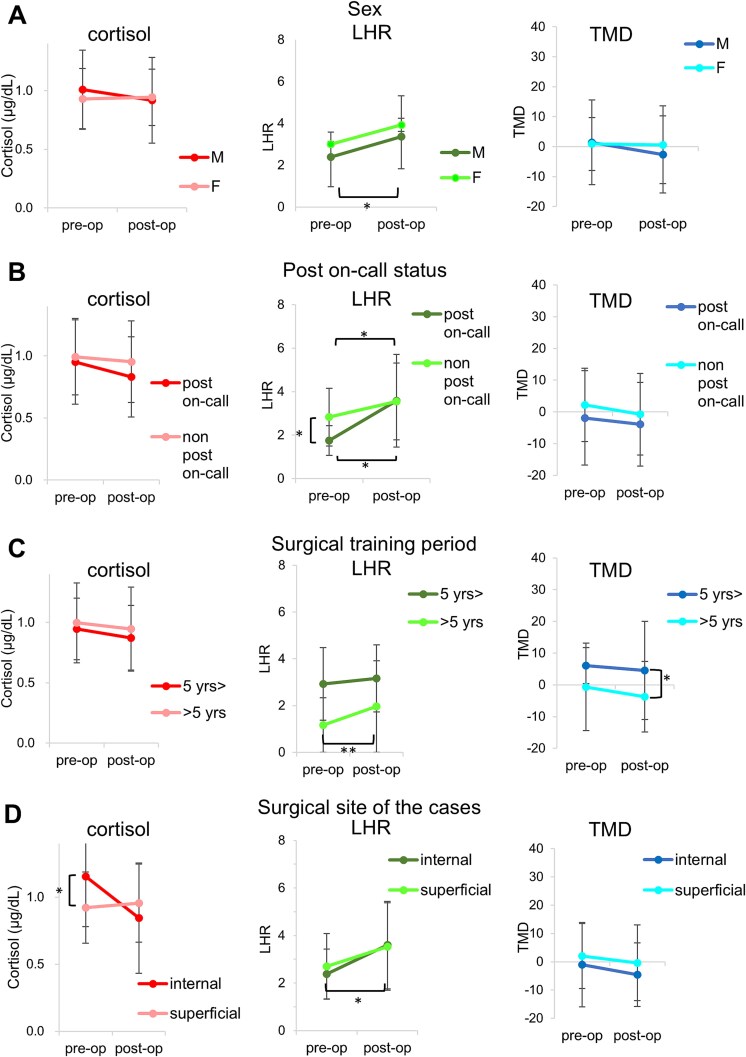
Changes in salivary cortisol, LHR, and TMD in the SW group based on differences by sex, on-call status, years of experience, and surgical site. (A) Comparison by sex. (B) Comparison by post-on-call status. ^*^P <0.05. (C) Comparison by surgical training period. ^*^P < .05, ^**^P < .01. (D) Comparison by surgical site of the cases. ^*^P < .05. LHR, low-/high-frequency heart rate ratio; SW, surgical working days; TMD, total mood disturbance.

Autonomic reactivity was assessed using the “My Beat” wearable monitoring device (Union Tool Co, Tokyo, Japan), which is worn continuously on the left chest, by calculating the low-to-high heart rate ratio (LHR).^7^^,^^10^ LHR represents the ratio of low-frequency to high-frequency heart rate components and reflects the balance between sympathetic and parasympathetic activity. The software provided by the manufacturer was used to determine LHR. A high LHR signifies sympathetic dominance, which is a state of tension, while a low LHR signifies parasympathetic dominance.^11^ Data were extracted, and the mean LHR values were visualized for the first and last 30 minutes of working hours in the NSW group, and for the period from 30 minutes before incision to 30 minutes after closure in the SW group (Figures 2B, 3, and  4). Moreover, for each day in the NSW and SW groups, all continuously monitored LHR values were plotted and their trends were analyzed (Figure 2D–G). The LHR trends were descriptively classified into 2 patterns based on visual assessment of the continuously monitored data: a “sustained elevation pattern,” in which elevated LHR persisted without clear recovery to baseline for a prolonged period, and a “baseline recovery pattern,” in which transient elevations were followed by repeated returns toward baseline levels. These classifications were exploratory and descriptive in nature.

### 2.4 POMS2

Participants’ moods were assessed using the POMS2, and the total mood disturbance (TMD) score was calculated. POMS2 is a self-report questionnaire developed to evaluate the subjective aspects of human emotions, such as mood and feelings, by measuring psychological distress at the time of response.^12^ It comprises 35 items rated on a 5-point Likert scale (0-4) and assesses 7 mood subscales: anger-hostility, confusion-bewilderment, depression-dejection, fatigue-inertia, tension-anxiety, vigor-activity, and friendliness. The TMD score was obtained by summing the scores of the 5 negative mood subscales (anger-hostility, confusion-bewilderment, depression-dejection, fatigue-inertia, and tension-anxiety) and subtracting the score of the positive mood subscale (vigor-activity). A higher TMD score indicates a more negative mood state; therefore, a decrease in TMD reflects mood improvement.^13^

### 2.5 Statistical analysis

The influence of sex, post-call status, and surgical training period on cortisol, LHR, and TMD was evaluated separately for NSWs and SWs using linear mixed-effects model analysis for repeated measurements in JMP Pro 17. Surgical site was included only in the SW model. To directly compare SWs and NSWs while accounting for repeated measurements obtained from the same surgeons, additional linear mixed-effects model analyses were performed. Workday type (NSW vs SW) and time point (pre vs post) were included as fixed effects, and participant ID was included as a random effect. Univariate analyses of each factor were performed successively. Each factor was independently compared with cortisol and LHR using the Kruskal-Wallis test, Spearman coefficient test, analysis of variance (ANOVA) test, and Student *t* test using GraphPad Prism 6.0, respectively. The association between 2 categorical variables in the 2 × 2 table was analyzed using Fisher exact or chi-square test. Differences were considered statistically significant at *P* < .05. Data were expressed as mean ± SD.

### 2.6 Ethical approval

Informed consent to participate in this study was obtained, a wearable device was attached, saliva samples were collected, and the POMS2 questionnaire was completed. This study was approved by the Institutional Review Board of Juntendo University School of Medicine (E21-0214) and complied with the Helsinki Declaration of 2008.

## 3. Results

### 3.1 Factors associated with cortisol, LHR, and TMD within SWs and NSWs

In this study, a total of 54 evaluation sessions for SWs and 44 evaluation sessions for NSWs were analyzed from 19 pediatric surgeons including 13 men and 6 women. The mean age was 33.8 ± 7.1 years (range, 27-50 years) (Table S1). The characteristics of the SW group were as follows: male-to-female ratio of 36:18, post-call/non-post-call ratio of 12:42, surgical training period of <5 or >5 years ratio of 14:40, and surgical site ratio (superficial to internal) of 38:16, including procedures such as inguinal hernia repair, circumcision, interval appendectomy, colostomy closure, pyeloplasty, and hepaticojejunostomy. The operative times for superficial and internal surgeries were 54 ± 23 and 280 ± 177 minutes, respectively. In contrast, the NSW group had a male-to-female ratio of 33:11, a post-call/non-post-call ratio of 15:29, and a surgical training period of <5 years or >5 years, with a ratio of 22:22 (Table S2). The SW and NSW groups showed no statistically significant differences in background characteristics except for the surgical training period (*P* < .05).

Linear mixed-effects model analyses revealed no significant associations between repeatedly measured cortisol and LHR values and post-call status, surgical training period, sex (for SW or NSW), or surgical site (only for SW) (Tables S3 and S4). Only TMD showed a significant association with the surgical training period in both the NSW group (95% CI, 3.01296-22.36261; *P* < 0.05) and SW group (95% CI, 1.07436-13.35229; *P* < .05), indicating a low TMD score (relatively positive mood state) in senior surgeons and experienced fellows (>5 years) in both SW and NSW groups.

### 3.2 Linear mixed-effects model analysis comparing SWs and NSWs

To directly compare SWs and NSWs while accounting for repeated measurements obtained from the same surgeons, an additional linear mixed-effects model analysis was performed with participant ID included as a random effect (Table 1). Salivary cortisol levels remained significantly higher on SWs than on NSWs (95% CI, −0.24207 to −0.00978; *P* < .05), whereas TMD scores remained significantly lower on SWs than on NSWs (95% CI, 2.36607-9.98148; *P* < .01). No significant difference in LHR was observed between SWs and NSWs.

**Table 1:** Linear mixed-effects model analysis comparing surgical and nonsurgical workdays.

						CI	
		Coefficient	Standard error	*t*	*P* value	95% lower	95% upper
**Cortisol**	NSW vs SW	−0.12593	0.05881	−2.14	.0338	−0.24207	−0.00978
	Pre vs post	−0.08313	0.05527	−1.50	.1344	−0.19224	0.02598
**LHR**	NSW vs SW	−0.02451	0.29071	−0.08	.92329	−0.59842	0.54939
	Pre vs post	0.48432	0.26150	1.85	.0659	−0.03231	1.00095
**TMD**	NSW vs SW	6.17377	1.92931	3.20	.0016	2.36607	9.98148
	Pre vs post	−0.82979	1.67089	−0.50	.6201	−4.12857	2.46900

Abbreviations: LHR, low/high heart rate ratio; TMD, total mood disturbance.

### 3.3 Comparison of salivary cortisol, LHR, and TMD between SW and NSW groups

Next, we compared changes in cortisol, LHR, and TMD between SW and NSW groups. Cortisol levels were relatively higher in the SW group both before and after work. Because the post-work decline was smaller on SWs, the levels remained significantly higher than those in the NSW group (*P* < .05) (Figure 2A). LHR significantly increased after work on SWs (*P* < .05), whereas it remained nearly unchanged on NSWs (Figure 2B). In contrast, TMD was lower on SWs than on NSWs, both before and after work (*P* < .05, *P* < .01) (Figure 2C). Interestingly, analysis of the continuously monitored LHR values across all days for both the SW and NSW groups revealed that the LHR value could be classified into 2 distinct types: a “sustained elevation pattern,” where the LHR remained consistently elevated in most plots and did not return to the baseline (Figure 2D,F), and a “baseline recovery pattern,” where LHR showed transient elevations followed by regular returns to the baseline (Figure 2E,G). The distribution of these patterns differed significantly between the groups. In the SW group, the sustained elevation pattern was dominant in 36/54 cases (baseline recovery pattern: 18/54). Conversely, the NSW group predominantly exhibited a baseline recovery pattern (36/44) with only 8/44 patients showing a sustained elevation pattern. This significant difference (*P* < .01) indicated a sustained state of sympathetic tension on SWs compared with NSWs (Figure 2D–G). In summary, although cortisol and LHR in the SW group indicated sustained physiological activation with sympathetic predominance, mood was better as reflected by the lower mood disturbance score.

### 3.4 Changes in salivary cortisol, LHR, and TMD in the NSW group based on differences by sex, on-call status, and years of experience

In the NSW group, no significant differences in cortisol levels, LHR, or TMD were observed between men and women (Figure 3A). When comparing the post-call status, although no statistical differences were found, both groups showed decreased cortisol levels before and after work, with post on-call participants having higher levels at both time points. No significant differences were observed in LHR or TMD (Figure 3B). Cortisol levels and LHR showed no significant differences when compared with the years of experience. In contrast, consistent with the linear mixed-effects model analysis, TMD was significantly higher in the group with a surgical training period <5 years than >5 years, both before and after work (*P* < .05, *P* < .01, respectively), indicating greater mood disturbance among younger fellows (Figure 3C).

### 3.5 Changes in salivary cortisol, LHR, and TMD in the SW group based on differences by sex, on-call status, years of experience, and surgical site

In the SW group, no significant differences were observed between males and females in cortisol levels or TMD before and after surgery (Figure 4A). LHR increased after surgery in both sexes; however, the increase was statistically significant only in males (*P* < .05) (Figure 4A).

Among participants with post on-call duties, preoperative LHR levels were lower (*P* < .05); however, LHR increased after surgery in both groups (*P* < .05) (Figure 4B). No clear differences were observed in cortisol or TMD according to post on-call status (Figure 4B). Interestingly, when stratified by years of surgical experience, LHR significantly increased after surgery in those with >5 years of training (*P* < .01). Although not statistically significant, LHR was relatively higher in the <5 years group both before and after surgery (Figure 4C). TMD was significantly higher in the <5 years group than in the >5 years group both before and after surgery, with a significant difference observed postoperatively (*P* < .05) (Figure 4C). When compared by surgical site, preoperative cortisol level was higher in internal surgeries (*P* < .05); however, because of a marked postoperative decline, no difference remained after surgery (Figure 4D). LHR increased in both groups, with a statistically significant increase in the internal group (*P* < .05) (Figure 4D). Although not statistically significant, TMD was lower in internal surgeries than in superficial surgeries, both before and after surgery (Figure 4D).

Collectively, these subgroup analyses suggest that post on-call status may be associated with a greater postoperative increase in LHR, and that internal surgeries may be associated with higher preoperative cortisol levels and postoperative increases in LHR, reflecting greater physiological activation. Moreover, a shorter surgical training period was associated with higher TMD on SWs, similar to the observations for NSWs. As we considered whether the larger proportion of participants with longer surgical training periods in the SW group might have influenced the TMD values, as shown in Table S2, we compared TMD between SW and NSW groups within each training-period group. Indeed, no difference in TMD values was observed among participants with a surgical training period >5 years (Figure S1A), whereas those with a surgical training period <5 years showed significantly lower TMD values in the SW group (Figure S1B). These findings suggest that the association between surgical work and reduced mood disturbance may be particularly pronounced among younger fellows.

## 4. Discussion

The present study demonstrated both physical and psychological strain, as evidenced by a smaller decline in cortisol levels on SWs and increased LHR after surgery, based on noninvasive measurements and quantitative analysis. A smaller decline in cortisol during SWs may indicate sustained physiological activation associated with operative performance or delayed recovery from task-related arousal rather than increased stress alone. Moreover, SWs were characterized by a dominant “sustained elevation pattern” of LHR, indicating a persistent state of sympathetic tension and sustained physiological activation compared with NSWs, which may reflect adaptive task-related arousal during operative performance. However, the TMD score remained low during surgical work, suggesting that a better mood state was maintained. These findings suggest that surgical work may involve coexistence of sustained physiological activation and favorable mood-state changes. Furthermore, a novel aspect of our findings lies in the clear results obtained using POMS2—a tool highly suitable for reflecting mood states that are susceptible to change based on personal behavior and the surrounding environment—for pre- and postoperative assessment and comparison with NSWs. These findings highlight the significance of working conditions for pediatric surgeons by incorporating mood disorder scoring and stress evaluation during surgery. Previous studies have reported that surgery can induce physiological stress responses such as increased heart rate and cortisol secretion.^4^^,^^14^ However, the postoperative decline in TMD observed in our study implies that surgeons may achieve adaptive psychological responses associated with operative task completion, reflecting professional satisfaction and self-efficacy. This aligns with recent reports emphasizing that surgeons’ well-being and professional fulfillment are closely linked to the intrinsic rewards of performing surgery and contributing to optimal patient outcomes.^15^^,^^16^ Several psychological frameworks further explain why surgeons’ moods improve despite physiological stress. According to the Job Demands–Resources model, high job demands can be counterbalanced by strong job resources such as autonomy, mastery, and intrinsic meaning, which contribute to positive mood-state changes and enhance work engagement.^17^ Moreover, the flow theory posits that performing highly skilled tasks under deep concentration evokes an intrinsically rewarding state that fosters positive affect.^18^

Another study reported occupational stress in pediatric surgeons.^19^ Gigola et al.^19^ assessed the presence and risk of burnout using a 56-item electronic questionnaire that included 22 items of the Maslach Burnout Inventory–Human Services Survey (MBI-HSS) specifically designed to evaluate work-related stress rather than general mood states such as those measured by POMS2. They demonstrated that working more than 50 h/wk, perceiving staff shortages, and being a resident were high-risk factors for burnout. Interestingly, limited opportunity to perform surgical procedures was identified as one of the stressors. In the present study, TMD scores were lower among surgeons who had more operative opportunities, which is consistent with the findings of Gigola et al.^19^ Unlike their study, the present study incorporated objective physiological measurements such as salivary cortisol and autonomic nervous activity, along with assessments before and after surgery, which adds a novel aspect to our investigation.

The physical and psychological burdens of post-on-call duties have been reported in various studies.^20^^,^^21^ In addition, a study of surgeons who had been on-call reported that elevated heart rates, indicative of sustained tension, persisted even 2 days after on-call duties.^6^ In our data, cortisol levels were elevated on NSWs following post-on-call, and LHR increased postoperatively on SWs. However, the effects of post-on-call duties were not clear in other measured parameters. In other words, mood disturbance after post-on-call duties remained relatively limited, suggesting that post-on-call duties may not pose a significant problem, as long as the safety and accuracy of medical practice are preserved. Nevertheless, this analysis could help identify individuals who experience difficulty after on-call work, for whom adjustments to work–life balance may be warranted.

Regarding the difference in years of surgical experience, the higher TMD scores among younger surgeons during NSWs may be due to factors such as a heavier workload than their seniors or an increased burden of nonclinical tasks.^22^ Similarly, TMD scores were higher in younger surgeons during SWs, which might reflect a psychological aspect in which more experienced surgeons operate with greater confidence and autonomy. Our data showing that LHR values were higher in younger surgeons are consistent with those of Weenk et al.,^4^ who demonstrated that intraoperative LHR via a wearable device monitor indicated lower stress levels in consultants than in young surgeons.

Regarding the difference in surgical sites, cortisol levels were higher in the internal group at the start of surgery, a finding considered a valid reflection of the tension associated with commencing the operation. Additionally, relatively lower TMD scores in the internal group might indicate that surgeons’ psychological adaptation is derived from performing technically demanding operations. Both of these factors introduce potential bias.

The present study has several limitations. First, this was a single-center exploratory observational study with a relatively small sample size. In particular, the subgroup analyses may have been underpowered and should therefore be interpreted cautiously. Second, the content of nonsurgical work and the specifics of surgical procedures were not standardized. Third, the time of saliva sampling at the end of surgery could not be consistently fixed. In general, cortisol shows a marked diurnal rhythm, characterized by a rapid rise after early morning, followed by a gradual decline throughout the daytime and afternoon.^23^^,^^24^ Because sample collection was performed outside the early morning peak cortisol period, the influence of circadian variation may have been smaller than that observed in early morning measurements. Nevertheless, the effect of sampling time cannot be excluded and should be considered when interpreting the cortisol findings. Additionally, the practical utility of cortisol measurements, LHR data, and POMS2 scoring for real-time monitoring of a surgical team’s stress levels in clinical settings is limited. Therefore, more rapid and feasible evaluation methods must be developed for future applications.

Based on the findings of the present study, the quantification of individual surgeons’ physical and psychological burdens and their mood-state changes may provide useful information when reevaluating practical elements such as daily routines, meeting frequency, and the structure of surgical or on-call teams. Furthermore, we believe that analyzing the impact of physiological activation and stress responses and variability on patient clinical outcomes is essential in the future. In conclusion, surgical work was associated with coexistence of sustained physiological activation and favorable mood-state changes among pediatric surgeons.

## Supplementary Material

Supplementary_materials_uiag036

## Data Availability

The data underlying this article are available in the article and in its online supplementary material.
