# Supplementary material for: Surgical paradox: physiological activation and improved mood state during surgical work in pediatric surgeons
Source: J Occup Health. 2026 Jul 4;68(1):uiag036. doi: 10.1093/joccuh/uiag036 (PMC13418209; doi:10.1093/joccuh/uiag036)
Supplement: Supplementary_materials_uiag036 [file supplementary_materials_uiag036.zip › Iida et al Supple Table June 25.docx]

**Supplementary Table 1 Participants’ sex distribution and age**

| Number of pediatric surgeons | 19 |
| --- | --- |
| Sex (Male/Female) | 13/6 |
| Age, mean ± SD (years) | 33.8 ± 7.1 |
| Age range (years) | 27-50 |

SD: standard deviation.

**Supplementary Table 2 Clinical characteristics of the study participants**

|  | Surgical work day (SW)  (n=54 tests) | Nonsurgical work day (NSW)  (n=44 tests) | *p* value |
| --- | --- | --- | --- |
| Sex  (Male/Female) | 36/18 | 33/11 | N.S. |
| After night duty  (post on-call / non-post on-call) | 12:42 | 15:29 | N.S. |
| Surgical training period  (<5 years or >5 years) | 14:40 | 22:22 | <0.05 |
| Surgical site of the cases (superficial / internal) | 38:16 | N/A | N/A |
| Operative time (min) (superficial or internal) | 54±23 280±177 | N/A | N/A |

SW: surgical workdays; NSW: nonsurgical workdays; N.S.: Not significant; N/A: not applicable.

**Supplementary Table 3 Linear mixed-effects model analysis of factors associated with nonsurgical working days**

|  |  | Coefficient | Standard error | t | *p* value | 95% lower | 95% upper |
| --- | --- | --- | --- | --- | --- | --- | --- |
| Cortisol | Female vs. Male | 0.13825 | 0.10825 | 1.28 | 0.2067 | -0.07844 | 0.35493 |
|  | Post-call (-) or (+) | -0.13248 | 0.11964 | -1.11 | 0.2727 | -0.37197 | 0.10700 |
|  | <5 vs. >5 years | 0.13714 | 0.09370 | 1.46 | 0.1487 | -0.05042 | 0.32470 |
| LHR | Female vs. Male | -0.11478 | 0.73591 | -0.16 | 0.8767 | -0.15936 | 1.36408 |
|  | Post-call (-) or (+) | -0.13311 | 0.78979 | -0.17 | 0.8669 | -0.17202 | 1.45404 |
|  | <5 vs. >5 years | 0.79776 | 0.63002 | 1.27 | 0.2114 | -0.46831 | 2.06383 |
| TMD | Female vs. Male | -4.72921 | 5.54505 | -0.85 | 0.3972 | -15.82484 | 6.36641 |
|  | Post-call (-) or (+) | 7.50082 | 6.23888 | 1.20 | 0.2341 | -4.98315 | 19.98480 |
|  | <5 vs. >5 years | 12.68778 | 4.83501 | 2.62 | 0.011 | 3.01296 | 22.36261 |

LHR: low/high heart rate ratio; TMD: total mood disturbance

**Supplementary Table 4 Linear mixed-effects model analysis of factors associated with surgical working days**

|  |  | Coefficient | Standard error | t | *p* value | 95% lower | 95% upper |
| --- | --- | --- | --- | --- | --- | --- | --- |
| Cortisol | Female vs. Male | -0.00786 | 0.08204 | -0.10 | 0.9239 | -0.17080 | 0.15508 |
|  | Post-call (-) or (+) | 0.11368 | 0.08229 | 1.38 | 0.1705 | -0.04975 | 0.27711 |
|  | <5 vs. >5 years | -0.00786 | 0.08204 | -0.71 | 0.4824 | -0.22172 | 0.10552 |
|  | Internal vs. Superficial | -0.06409 | 0.08689 | -0.74 | 0.4626 | -0.23667 | 0.10848 |
| LHR | Female vs. Male | 0.50264 | 0.42840 | 1.17 | 0.2438 | -0.34845 | 1.35373 |
|  | Post-call (-) or (+) | 0.49521 | 0.42635 | 1.16 | 0.2485 | 0.35180 | 1.34222 |
|  | <5 vs. >5 years | -0.37195 | 0.41746 | -0.89 | 0.3753 | -1.20131 | 0.45741 |
|  | Internal vs. Superficial | 0.02138 | 0.43391 | 0.05 | 0.9608 | -0.84065 | 0.88342 |
| TMD | Female vs. Male | -2.97126 | 3.05696 | -0.97 | 0.3336 | -9.04093 | 3.09841 |
|  | Post-call (-) or (+) | 2.06554 | 3.02466 | 0.68 | 0.4964 | -3.94000 | 8.07108 |
|  | <5 vs. >5 years | 7.21332 | 3.09186 | 2.33 | 0.0218 | 1.07436 | 13.35229 |
|  | Internal vs. Superficial | 2.43255 | 3.35567 | 0.72 | 0.4703 | -4.23021 | 9.09530 |

LHR: low/high heart rate ratio; TMD: total mood disturbance
