# Supplementary figures and images for: Surgical paradox: physiological activation and improved mood state during surgical work in pediatric surgeons
Source: J Occup Health. 2026 Jul 4;68(1):uiag036. doi: 10.1093/joccuh/uiag036 (PMC13418209; doi:10.1093/joccuh/uiag036)

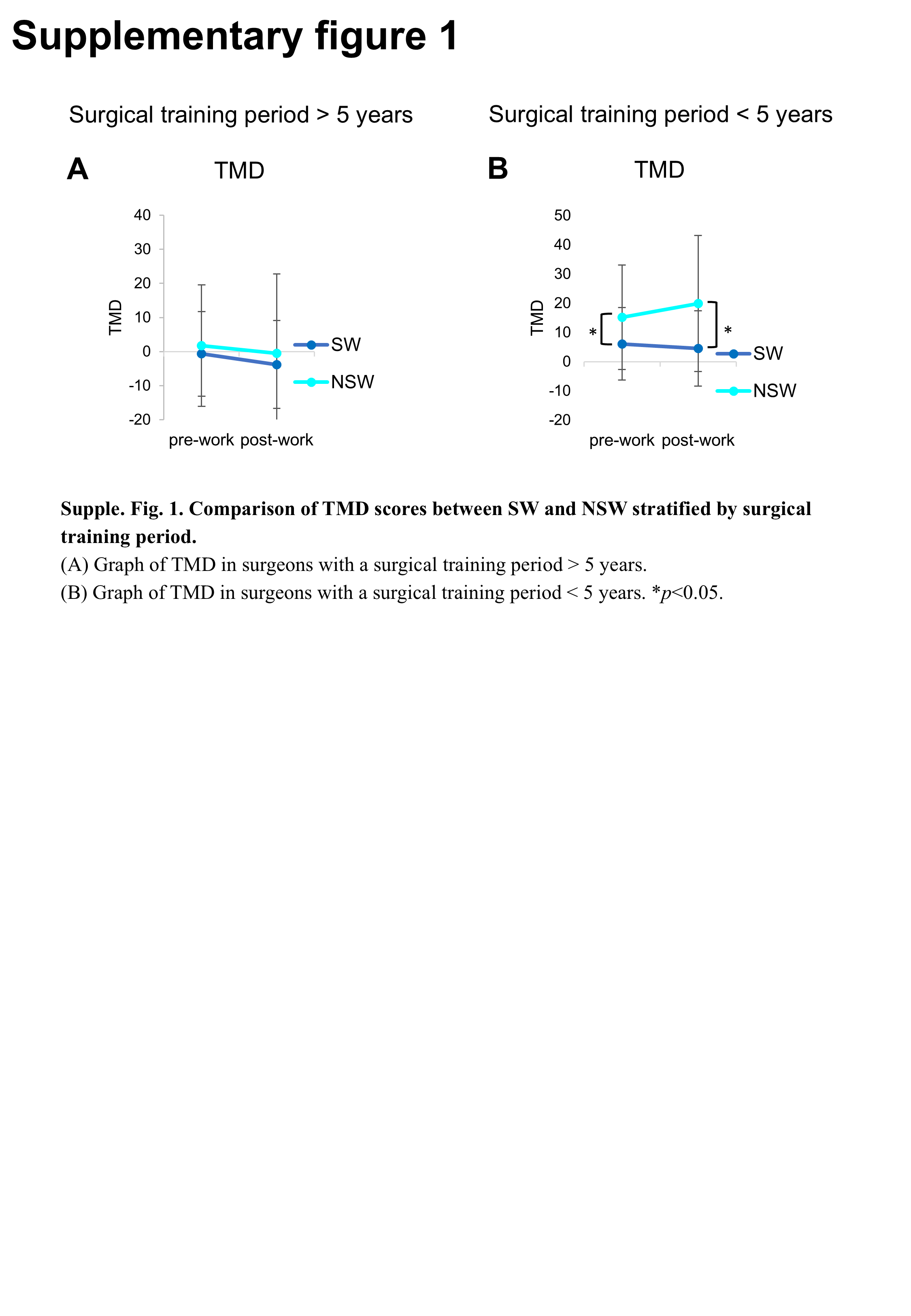

Supplement: Supplementary_materials_uiag036 [file supplementary_materials_uiag036.zip › SuppleFigure 1.TIF]
